# Multiplex TaqMan^®^ Quantitative PCR Assays for Host-Tick-Pathogen Studies Using the Guinea Pig-Tick-*Rickettsia* System

**DOI:** 10.3390/pathogens11050594

**Published:** 2022-05-18

**Authors:** Anne-Marie L. Ross, John V. Stokes, Claire E. Cross, Navatha Alugubelly, Andrea S. Varela-Stokes

**Affiliations:** Department of Comparative Biomedical Sciences, College of Veterinary Medicine, Mississippi State University, Mississippi State, MS 39762, USA; ar2324@msstate.edu (A.-M.L.R.); claire.e.cross@vanderbilt.edu (C.E.C.); navatha@attentivescience.com (N.A.)

**Keywords:** spotted fever rickettsiosis, *Amblyomma maculatum*, *Amblyomma americanum*, *Rickettsia parkeri*, *Rickettsia amblyommatis*, *Cavia porcellus*

## Abstract

Spotted Fever Rickettsiosis (SFR) is caused by spotted fever group *Rickettsia* spp. (SFGR), and is associated with symptoms common to other illnesses, making it challenging to diagnose before detecting SFGR-specific antibodies. The guinea pig is a valuable biomedical model for studying Spotted Fever Rickettsiosis (SFR); its immune system is more like the human immune system than that of the murine model, and guinea pigs develop characteristic clinical signs. Thus, we have a compelling interest in developing, expanding, and optimizing tools for use in our guinea pig-*Amblyomma*-*Rickettsia* system for understanding host-tick-pathogen interactions. With the design and optimization of the three multiplex TaqMan^®^ qPCR assays described here, we can detect the two SFGR, their respective primary *Amblyomma* sp. vectors, and the guinea pig model as part of controlled experimental studies using tick-transmission of SFGR to guinea pigs. We developed qPCR assays that reliably detect each specific target down to 10 copies by producing plasmid standards for each assay target, optimizing the individual primer-probe sets, and optimizing the final multiplex reactions in a methodical, stepwise fashion. We anticipate that these assays, currently designed for in vivo studies, will serve as a foundation for optimal SFGR detection in other systems, including fieldwork.

## 1. Introduction

Several spotted fever group *Rickettsia* species (SFGR) are agents of spotted fever rickettsiosis (SFR) in the United States. These include *R. rickettsii*, the agent of Rocky Mountain spotted fever (RMSF), and two emerging tick-borne agents, *Rickettsia* sp. 364D and *R. parkeri*, which cause SFR milder than RMSF. *Rickettsia* sp. 364D is transmitted primarily by *Dermacentor occidentalis* (Pacific Coast tick), and is located along the Pacific Coast [1,2]. *Rickettsia parkeri* is primarily transmitted by Gulf Coast ticks (*Amblyomma maculatum*), which are found predominantly in the southeastern United States, and can have approximately 40% *R. parkeri* infection rates in comparison to less than 0.1% for *R. rickettsii* in *Dermacentor* vectors [3,4]. *Rickettsia parkeri* was first identified as an agent of SFR in 2002 [5]. An SFGR that is considered nonpathogenic is *Rickettsia amblyommatis*, with tick infection rates exceeding 40% in some areas, and the lone star tick (*Amblyomma americanum*) vector, having a geographic distribution that overlaps with the Gulf Coast tick [6,7]. Past serological studies have shown cross-reactivity among SFGR, suggesting that humans are also exposed to less virulent and nonpathogenic SFGR, potentially affecting the number of SFR diagnoses made using seroassays [8]. When Blanton, et al., [9] demonstrated that prior exposure to the comparatively nonpathogenic SFGR, *R. amblyommatis*, provided clinical protection against lethal *R. rickettsii* exposure in guinea pigs, they also found that *Rickettsia amblyommatis*-exposed guinea pigs produced antibodies against both *R. amblyommatis* and *R. rickettsii* prior to challenge, confirming that cross-reactivity occurs, though its importance in cross-protection is unknown. Clarifying the role of low-pathogen SFGR in protection from SFGR with higher pathogenicity, using the guinea pig model, requires distinguishing the SFGR serologically and through molecular detection.

The guinea pig (*Cavia porcellus*), a valuable model for studying SFR, was first used in SFR research over 100 years ago to demonstrate clinical signs with *R. rickettsii* [10]. Guinea pigs have multiple advantages over the murine model, many of which have been previously reviewed [11,12]. Our guinea pig-*Amblyomma*-*Rickettsia* system uses molecular and immunological assays, such as qPCR, flow cytometry, and ELISA, to monitor tick-transmitted rickettsial infection. While the flow cytometric assay follows immunological responses during an active infection, qPCR can confirm a current infection, and is useful for specifically detecting rickettsial DNA when surveilling ticks from the field, and the enzyme-linked immunosorbent assay (ELISA) is useful for assessing past infections. Further, multiplex TaqMan^®^ qPCR assays are invaluable due to their ability to amplify multiple gene targets simultaneously.

The three optimized multiplex assays described here were designed for controlled studies to detect: (1) *R. parkeri* (Rp), *R. amblyommatis* (Ramb), and guinea pig (GP); (2) *R. parkeri*, *R. amblyommatis*, and lone star tick (LST); and (3) *R. parkeri*, *R. amblyommatis*, and Gulf Coast tick (GCT). While they have their limitations, these assays should prove useful for experimental infections using tick transmission to the guinea pig biomedical model.

## 2. Results and Discussion

### 2.1. Rp, Ramb, GP 3-plex

The primer and probe sequences and optimized concentrations for this assay are listed in Table 1. Initially, during the development of the 3-plex Ramb, Rp, GP assay, the efficiency of the individual standard curve for the guinea pig primer-probe set was consistently too low at ~85%. After several attempts to further optimize the probe and primer concentrations, we retired our original GAPDH-specific GP primers and probe and selected new GP primers universal to mammals, in addition to a GP probe specific to the guinea pig 12S rRNA gene. We then made a new plasmid standard, repeated the first three optimization steps, and tested the new primer-probe set with Ramb and Rp in the 3-plex in the final two optimization steps. The three standard curve replicates demonstrated that the primer-probe sets worked together well, as they all exhibited amplification efficiencies between 90% and 110%, and R2 values equal to or greater than 0.985 (Supplementary Figure S1).

### 2.2. Rp, Ramb, LST 3-plex

The primer and probe sequences and optimized concentrations for this assay are listed in Table 2. The LST primer-probe set initially showed the lowest efficiency of the three targets, hovering in the low 90% range when combined with Rp and Ramb. We increased the LST probe concentration from 200 nM to 400 nM, attempting to improve its efficiency. As the LST primer-probe set was still sometimes less than 90% efficient, we felt there was likely some competition or inhibition with the other primers or probes in the assay. Therefore, we decreased the Ramb probe from 200 nM to 50 nM and the Rp probe from 400 nM to 300 nM to decrease potential interference. These final optimization adjustments resulted in three consecutive runs, with three replicates on different days, with in-range amplification efficiency (90–110%) and R2 values (≥0.985) (Supplementary Figure S2).

### 2.3. Rp, Ramb, GCT 3-plex

The primer and probe sequences and optimized concentrations for this assay are listed in Table 3. Here, we initially attempted to design a 4-plex assay to include *Candidatus* Rickettsia andeanae (CaRa) as an additional nonpathogenic *Rickettsia* spp. to detect along with Rp and Ramb. Using the ompB gene region to produce the CaRa primers and probe, this target had cross-reactivity issues with Rp in the final optimization steps. After testing three different CaRa primer and probe sets to address this issue, we determined that its ompB sequence was too similar to Rp, and we could not differentiate the two species. Since CaRa did not elicit an immune response like Rp and Ramb, and the assay was being designed for controlled experimental studies rather than fieldwork, we elected to drop it as a target and keep the assay as a 3-plex. With no other issues, the combined standard curve replicates of this 3-plex were successful with no further adjustments (Supplementary Figure S3).

It is important to use a methodical stepwise approach when developing and optimizing complex assays. This development-optimization strategy is essential with multiplex qPCR assays since there are several targets, each requiring different primer-probe sets which can potentially interact with one another. At the outset, we determined our standard of what defined an optimized assay. Our criteria were that for three consecutive runs, each with three replicates, performed on different days, the amplification efficiencies for all targets must be between 90–110%, with R^2^ values ≥ 0.985%, with the singleplex and multiplex efficiencies not differing by more than ~5%, and with Cq values not varying by more than 1. Any assay that did not meet the criteria during the individual or combined standard curve steps required reassessing the primer or probe concentrations and adjusting accordingly. The primer and probe concentrations were chosen based on balancing a relatively low Cq with a relatively high ΔRn. This strategy of reducing the amount of reagent, where possible, decreased the potential of competitive or inhibitory reactions between primer-probe sets, and conserved resources.

### 2.4. Additional Testing

Once optimized using plasmid standards, we applied the assays to test genomic DNA from representative samples that would be available in an experimental study. We used the GCT and LST assays to test whole-tick DNA extractions, to assess efficacy and specificity with authentic samples. For the GCT assay, we used *R. parkeri* positive and negative GCT extracts as determined by prior qPCR experiments using a previously published assay (Lee, et al., 2017). Both samples were positive for the GCT target sequence, with Cq values of 20.71 and 21.18, respectively, whereas only the Rp positive sample was positive for the Rp sequence. The Ramb target sequence was not detected in either sample. Similarly, for the LST assay, *R. amblyommatis* positive and negative LST extracts were positive for the LST target sequence, at Cq values of 21.73 and 22.17, respectively. Only the extract from the LST sample that had been determined positive for the Ramb target sequence using a previously published assay, was positive in our assay (Lee, et al., 2017). Neither sample was positive for the Rp target sequence. We also cross-tested a GCT Rp-positive and a GCT Rp-negative DNA extraction on the LST assay, with an LST positive sample as a control. Neither GCT sample was picked up, but the Rp positive was still picked up by its specific target with a Cq of 19.85. Vice versa, we ran an LST Ramb positive and LST Ramb negative extraction sample on the GCT assay in the same way; likewise, neither LST sample showed on the GCT assay, but the Ramb positive sample had a Cq of 34.34, demonstrating that the Rp and Ramb were still distinguishable from off-species samples in both assays. In summary, the experiments for GCT and LST were able to discriminate between the different tick species and *R. parkeri* positive versus *R. amblyommatis* positive samples.

To further assess Rp and Ramb differentiation, we tested DNA extracts from Vero and ISE6 cell cultures infected with Rp, Ramb, and uninfected. Only the extracts from the Rp-infected and Ramb-infected cultures amplified their respective targets, while extracts from uninfected cell cultures had no Cq for any target.

In addition, we tested genomic DNA from *R. akari* (Bronx), *Rickettsia* spp. 364D (D03), and *R. rickettsii* (Sheila Smith) (kindly provided by Dr. Chris Paddock, Centers for Disease Control and Prevention). While the assays did not cross-react with the *R. akari* DNA, they showed amplification of *Rickettsia* spp. 364D and *R. rickettsii*, with Cq values of ~20 with the Rp primer-probe sets for both species and ~32 with the Ramb primer-probe sets for both species, confirming that the assays should not be used with extracts from ticks of unknown geographical location, from ticks that are not laboratory-reared, or from ticks that were not previously screened for these other SFGR.

Finally, while we designed these assays for detection, they have potential and planned utility for quantification. Before use for quantification, the assays will also be tested using mixtures of plasmid standards at varying known copy numbers compared to plasmid standard mixtures with equal copy numbers.

## 3. Materials and Methods

### 3.1. Vertebrate Sample Collection

We extracted DNA from a guinea pig ear punch for the template to produce a control plasmid for the guinea pig qPCR target. All procedures were conducted with approval from the Mississippi State University Institutional Animal Care and Use Committee (IACUC), following AAALAC guidelines (IACUC protocol numbers 17-166 and 18-267).

### 3.2. Primer and Probe Design

We used the Custom Design service through Integrated DNA Technologies (IDT; Coralville, Iowa) to design primers and probes for *R. amblyommatis* and *R. parkeri*, with the same primer and probe set used in all assays, at varying concentrations (Table 1, Table 2 and Table 3). Briefly, we used GenBank accession numbers KX151487.1 (*R. amblyommatis* strain Ac/Pa, ompB, partial sequence) and AF123717.1 (*R. parkeri* strain Maculatum 20, ompB, partial sequence) to align sequences and identify primers and probes with optimal Tm values for specific amplification of 108-bp and 103-bp amplicons of *R. amblyommatis* and *R. parkeri*, respectively. As *R. amblyommatis* KX151487.1 was amplified from *A. cajennense* and not *A. americanum*, we proceeded to confirm 100% identity with a representative *Candidatus* R. amblyommii ompB sequence (JN378402.1) from *A. americanum* within the region of the selected primer/probe set, using SnapGene^®^ V6 software (Table 1). The *R. amblyommatis* primer set also recognized ompB sequences from those submitted as *R. amblyommii* (synonymous to *R. amblyommatis*), *Rickettsia* sp. WB-8-2, a strain of *R. amblyommatis*, and a closely related strain *Rickettsia* sp. MOAa. Rickettsial primer/probe sets did not amplify gene sequences from the tick vectors, *Amblyomma americanum* and *Amblyomma maculatum*, as confirmed by IDT during design and after conducting a BLAST of the primers and probes against *Amblyomma* spp. sequences.

We based amplification of the guinea pig 12S rRNA gene target on published mammalian-wide primers, and modified the primers to adjust Tm; Universal 12S-F was modified to remove the first three base pairs, and Universal 12S-R was modified to remove the last seven base pairs to match the Tm values and allow for an appropriate probe with Tm 5 °C higher (60 °C) than that of the primers [14]. The 12S rRNA probe was designed using SnapGene^®^ software.

We used the primers and probes described above to amplify the *R. amblyommatis* and *R. parkeri* qOmpB gene target in lone star ticks. For primers and probes to detect the lone star tick macrophage migration inhibitory factor (*MIF*) gene, we used the Custom Design service through Integrated DNA Technologies (IDT; Coralville, IA, USA), based on the sequence from *A. americanum* GenBank accession number AF289543.2. The target was 102 base pairs.

To detect SFGR in Gulf Coast ticks, we amplified the qOmpB gene target in *R. parkeri* and *R. amblyommatis*, with *R. amblyommatis* serving primarily as an internal negative control, since this SFGR has not been detected in Gulf Coast ticks to our knowledge.

### 3.3. Plasmid Standards

Plasmids containing cloned target gene sequences were produced in-house for use as qPCR standards. We generated the target insert for each plasmid standard using genomic DNA extracts known to be positive for the gene of interest, as a template. Archived Gulf Coast and lone star tick DNA extracts were selected from previous studies; guinea pig genomic DNA was extracted from ear punches; and *R. parkeri* (Portsmouth) and *R. amblyommatis* (Line Creek) DNA was extracted from co-cultures of rickettsiae with Vero cells. Specific sequences were first amplified by conventional PCR using the relevant primers for each plasmid standard. Then, the predicted amplicon length was confirmed by agarose gel electrophoresis using a 100-bp ladder and SYBR green staining. Next, the amplified PCR insert was cloned into a pCR 4-TOPO vector from an Invitrogen TOPO TA Cloning Kit, and OneShot TOP10 competent E. coli cells were transformed with the plasmid. The transformed cells were plated on LB-agar plates with 100 µg/mL ampicillin, and incubated overnight at 37 °C. We picked six colonies to analyze by conventional colony PCR followed by gel electrophoresis to confirm specificity and base-pair length of the plasmid insert. Finally, from the colonies that produced an amplicon of the correct size, we chose a single colony from which to prepare overnight cultures for purifying the plasmid DNA by miniprep. We used a Nanodrop spectrophotometer to determine the plasmid purity, and a Qubit fluorometer to determine the concentration. The plasmid was aliquoted at a final concentration of 10^8^ copies per µL, to be used as a qPCR standard. All three multiplex assays had a 5 μL plasmid mix made as the standard template to add to the reaction master mix. The plasmid mix volume for each assay consisted of 1 μL from the final plasmid concentration for each target and molecular grade water to Q.S. the volume to 5 μL. Thus, each 3-plex assay had 3 μL of plasmid template and 2 μL of water. For a complete list of resources, see Supplementary Table S1.

### 3.4. Assay Optimization

**Figure 1:**
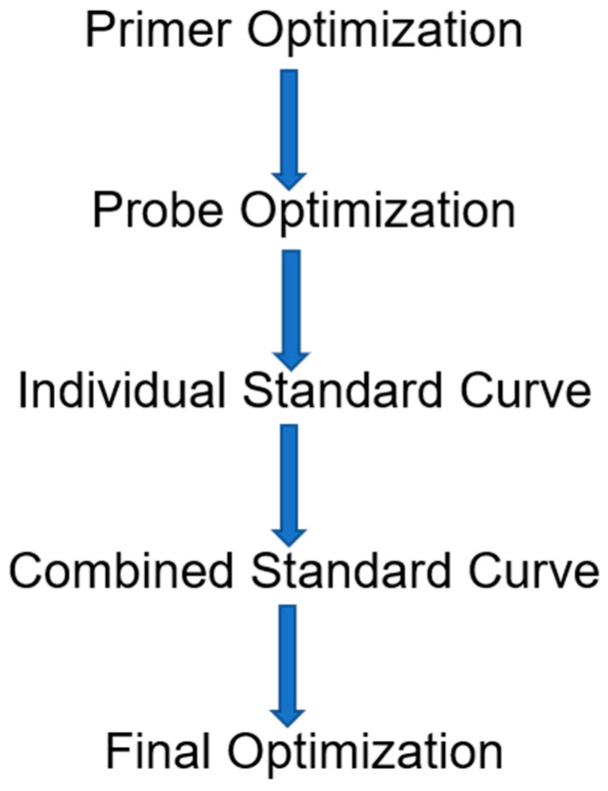
The sequential strategy utilized for the Taqman qPCR assay optimizations.

**Figure 2:**
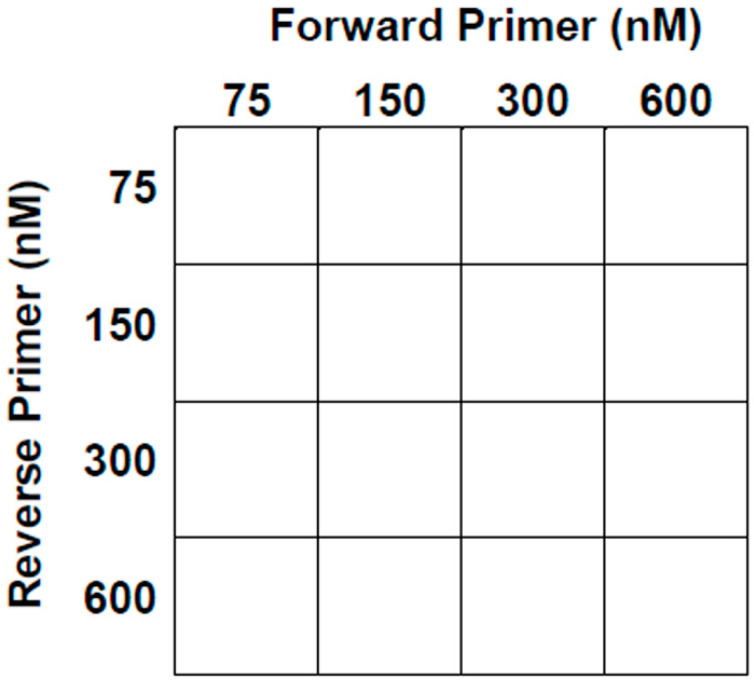
The primer optimization step utilized this matrix design to determine the optimal combination of concentrations.

Once our primers, probes, and plasmid standards were designed and prepared, we optimized each assay in a five-step sequence (Figure 1). First, we determined the optimal primer concentrations by analyzing 16 different forward and reverse primer-pair concentration combinations, each with six replicates; during this step, we used a constant probe concentration of 200 nM to determine which combination resulted in the lowest Cq (Figure 2). The assay was conducted using Agilent Brilliant Multiplex QPCR Master Mix with a ROX reference dye and a plasmid quantity of 10^5^ copies. If two or more primer concentration combinations resulted in a similar Cq, we chose the lowest concentration with the higher or comparable ΔRn. The ΔRn was the baseline-corrected and normalized fluorescence. Then, we determined the optimal probe concentration. This assay was completed using the optimal primer pair from the first step. Similarly to the first step, we evaluated four probe concentrations, 50 nM, 100 nM, 200 nM, and 400 nM, and again we initially chose the concentration that resulted in the lowest Cq. As with the primer-pair selection, if two or more probe concentrations resulted in a similar Cq, we selected the lowest concentration with a higher, or comparable, ΔRn value.

After determining the optimal primer and probe concentrations, standard curves prepared from serial dilutions of 10^7^ to 10^1^ plasmid copies were analyzed for each primer-probe set in triplicate, to assess the efficiency and linearity of the single reaction. No template controls (NTC) of molecular grade water were included on the plate to control for extraneous nucleic acid contamination. Then, we assessed standard curves that included all the primer-probe sets and plasmid standards for each of the multiplex assays under the same conditions as the single primer-probe sets. The final optimization step for each of the assays consisted of making any minor concentration adjustments needed, based on the results of the combined standard curve. Our criteria for an optimized assay were that, for over three consecutive runs, each with three replicates, performed on different days, the amplification efficiencies for all targets must be between 90–110%, with R^2^ values ≥ 0.985%; further, the singleplex and multiplex efficiencies must not differ by more than ~5%, and Cq values must not differ by more than 1. The thermal cycling was the same for all plates, with a hot start at 95 °C for 10 min and 40 cycles of amplification at 95 °C for 15 s and 60 °C for 1 min. For a complete list of resources, see Supplementary Table S1.

### 3.5. Data Acquisition and Analysis

We analyzed all plates with an Agilent AriaMx Real-Time PCR instrument and the Agilent AriaMx Software Version 1.71 (Agilent Technologies, Inc. Santa Clara, CA, USA). For a complete list of resources, see Supplementary Table S1.

## 4. Conclusions

The three TaqMan^®^ multiplex qPCR assays effectively differentiate all DNA targets and rickettsial species detection down to at least 10 copies in controlled experiments using the guinea pig model for spotted fever rickettsiosis, where the rickettsial species are known to be either *R. parkeri* or *R. amblyommatis*, and their primary vectors, *A. maculatum* and *A. americanum*, respectively, are used. Thus, the principal limitation of the assays is the lack of efficacy for field studies. As we continue to develop the guinea pig model for spotted fever rickettsiosis, qPCR assays that distinguish tick-transmitted rickettsiae known to cause disease (*R. parkeri*) or common in an overlapping tick species (*R. amblyommatis*) will provide data on infection dynamics in the animal model, and will monitor the infection status of the respective tick vectors.

## Figures and Tables

**Table 1:** Primers and probes for 3-plex qPCR assay targeting *R. parkeri*, *R. amblyommatis*, and guinea pig. The *R. parkeri* probe was labeled with HEX, the *R. amblyommatis* probe with FAM, and the guinea pig probe with CY5.

Target	Primer/Probe	Concentration (nM)	Sequence (5′–3′)
*R. parkeri ompB*	qOmpB_Rp_F	300	CGT GAC GGT GAT GTT GCT ATT A
	qOmpB_Rp_R	600	CGG CAG CAT TTG TAG TTC TTG
	qOmpB_Rp_p	400	/5HEX/AAC GGT GCA/ZEN/GTA CAA TTC GCT CAT/3IABkFQ/
*R. amblyommatis ompB*	qOmpB_Ramb_F	150	AAA GCA CCA CCG ACA ACA
	qOmpB_Ramb_R	300	ACA TAC TGC CGA GTT ACG TTT AG
	qOmpB_Ramb_p	200	/56-FAM/ACC GTT TAT/ZEN/AAC TGT GCC GTC AGC A/3IABkFQ/
*Guinea pig 12S rRNA*	Universal 12S-F	150	ACC GCG GTC ATA GCA TT
	Universal 12S-R	300	GGG TAT CTA ATC CCA GTT TGG G
	Cavia 12S-p	200	/5Cy5/AGT TAA TAA/TAO/ACC CCG GCG TAA AAA GTG/3IAbRQSp/

**Table 2:** Primers and probes for the 3-plex qPCR assay targeting *R. amblyommatis*, *R. parkeri*, and *lone star tick*. *R. parkeri* and *R. amblyommatis* probes were labeled as in Table 1, with the lone star tick labeled with CY5.

Target	Primer/Probe	Concentration (nM)	Sequence (5′–3′)
*R. parkeri ompB*	qOmpB_Rp_F	300	
	qOmpB_Rp_R	600	All sequences as in Table 1
	qOmpB_Rp_p	300	
*R. amblyommatis* *ompB*	qOmpB_Ramb_F	150	
	qOmpB_Ramb_R	300	All sequences as in Table 1
	qOmpB_Ramb_p	50	
*Lone star tick MIF*	LST-MIFf	75	CGA ATC GTC TCT GCG TCT TT
	LST-MIFr	300	TTT GCA GCG TTG AGA AAG TAT G
	LST-MIFp	400	/5Cy5/TGA GTG CGA/TAO/TTT CCG TAC AGA GCA/3IAbRQSp/

**Table 3:** Primers and probes for the 3-plex qPCR assay targeting *R. parkeri*, *R. amblyommatis*, and *Gulf Coast tick*. The *R. parkeri* and *R. amblyommatis* probes were labeled as in Table 1, with the *Gulf Coast tick* probe labeled with CY5.

Target	Primer/Probe	Concentration (nM)	Sequence (5′–3′)
*R. parkeri ompB*	qOmpB_Rp_F	300	
	qOmpB_Rp_R	600	All sequences as in Table 1
	qOmpB_Rp_p	400	
*R. amblyommatis ompB*	qOmpB_Ramb_F	150	
	qOmpB_Ramb_R	300	All sequences as in Table 1
	qOmpB_Ramb_p	200	
*Gulf Coast tick MIF*	AmacMIF.18F	150	CCA GGG CCT TCT CGA TGT [13]
	AmacMIF.99R	300	CCA TGC GCA ATT GCA AAC C [13]
	AmacMIF.63	200	TGT TCT CCT TTG GAC TCA GGC AGC [13]

## Data Availability

Not applicable.

## Supplementary Materials

The following supporting information can be downloaded at: https://www.mdpi.com/article/10.3390/pathogens11050594/s1, Figure S1 (A–D): Primer and probe optimization values for Rp, Ramb, GP 3-plex; Figure S2 (A–D): Primer and probe optimization values for Rp, Ramb, LST 3-plex; Figure S3 (A–D): Primer and probe optimization values for Rp, Ramb, GCT 3-plex; Table S1: Complete list of reagents and resources.
