# Supplementary material for: Multiplex TaqMan® Quantitative PCR Assays for Host-Tick-Pathogen Studies Using the Guinea Pig-Tick-Rickettsia System
Source: Pathogens. 2022 May 18;11(5):594. doi: 10.3390/pathogens11050594 (PMC9147651; doi:10.3390/pathogens11050594)
Supplement: Supplementary file 1 [file pathogens-11-00594-s001.zip › pathogens-1727790-supplementary.pdf]

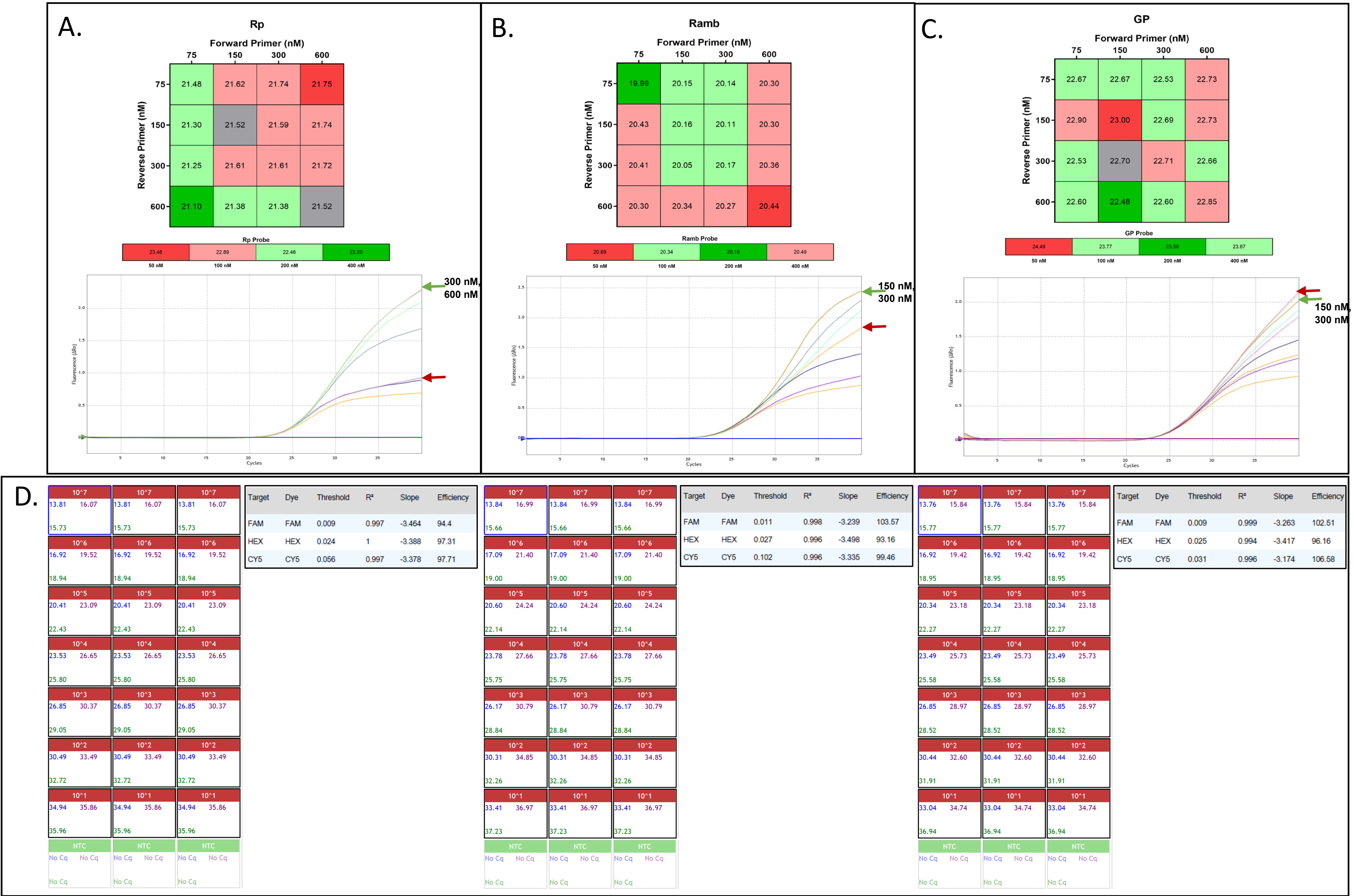

Figure S1.(A). The *R. parkeri* primer and probe optimization matrices (top) illustrate the optimal and lowest Cq (dark green) and highest Cq (dark red). Mean Cq values are gray. In the amplification curve, the optimal Rp primer concentration (300 nM, 600 nM) had a low Cq with a high ΔRn (green arrow); red arrow denotes primer concentrations that only had lowest Cq.

Figure S1.(B). The *R. amblyommatis* primer and probe optimization matrixes (top) illustrate the optimal and lowest Cq (dark green) and highest Cq (dark red). In the amplification curves, the optimal Ramb primer concentration (150 nM, 300 nM) had a low Cq with a high ΔRn (green arrow); red arrow denotes primer concentrations that only had lowest Cq.

Figure S1.(C). The guinea pig primer and probe optimization matrices (top) illustrate the optimal and lowest Cq (dark green) and highest Cq (dark red). Mean Cq values are gray. In the amplification curves, the optimal GP primer concentration (150 nM, 300 nM) had a low Cq with a high ΔRn (green arrow); red arrow denotes primer concentrations that only had lowest Cq.

Figure S1.(D). Average Cq value for each target from concentrations of 10^7 to 10^1 of plasmids from HEX-labeled *R.parkeri* (green), FAM-labeled *R. amblyommatis* (blue) and CY5-labeled GP (purple). Three plates tested in triplicates, shown here with efficiencies for each respective run next to image of Cq values.

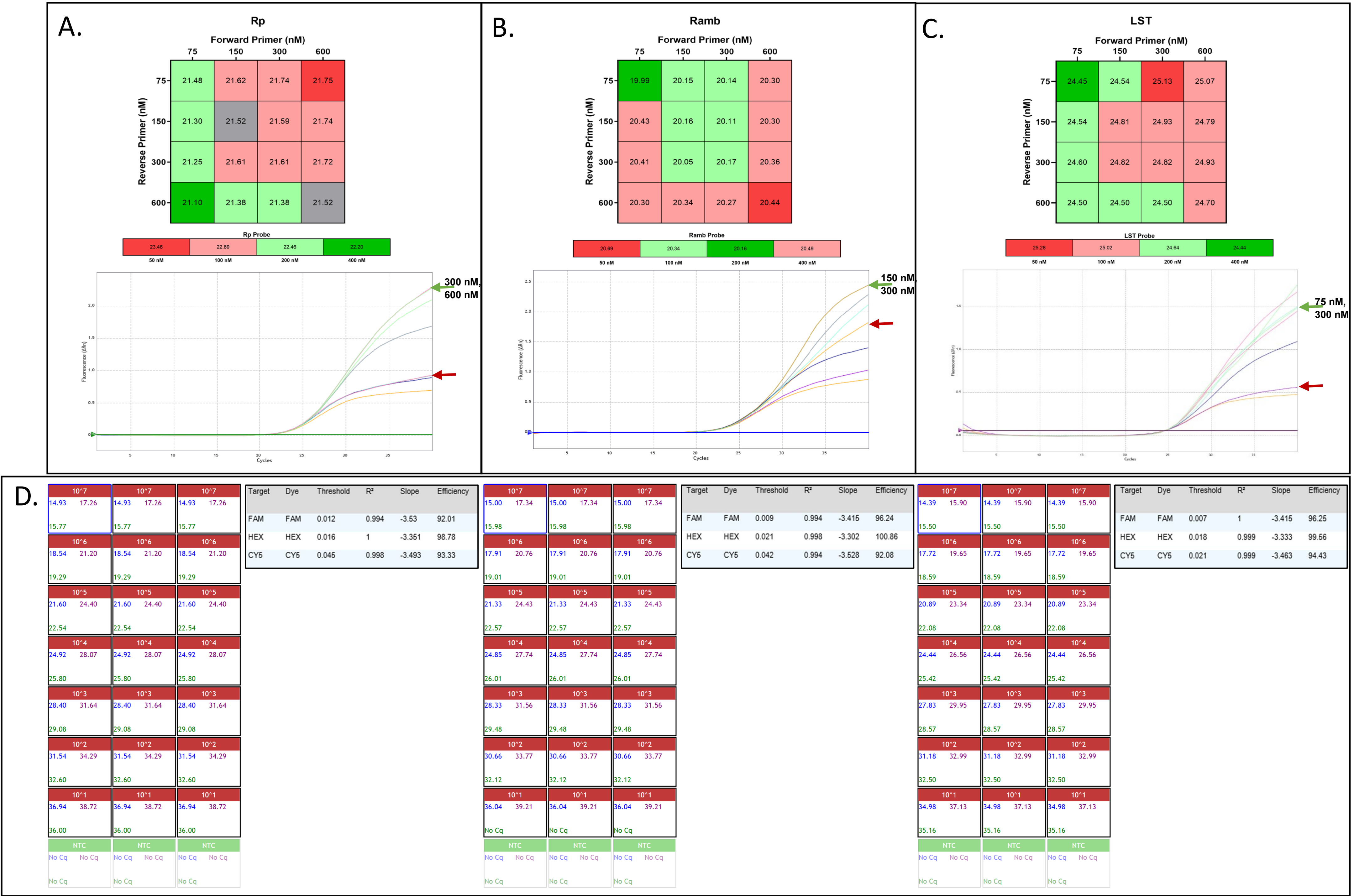

Target	Dye	Threshold	R²	Slope	Efficiency
FAM	FAM	0.012	0.994	-3.53	92.01
HEX	HEX	0.016	1	-3.351	98.78
CY5	CY5	0.045	0.998	-3.493	93.33

Target	Dye	Threshold	R²	Slope	Efficiency
FAM	FAM	0.009	0.994	-3.415	96.24
HEX	HEX	0.021	0.998	-3.302	100.86
CY5	CY5	0.042	0.994	-3.528	92.08

Target	Dye	Threshold	R²	Slope	Efficiency
FAM	FAM	0.007	1	-3.415	96.25
HEX	HEX	0.018	0.999	-3.333	99.56
CY5	CY5	0.021	0.999	-3.463	94.43

Figure S2.(A). The *R. parkeri* primer and probe optimization matrices (top) illustrate the optimal and lowest Cq (dark green) and highest Cq (dark red). Mean Cq values are gray. In the amplification curves, the optimal Rp primer concentration (300 nM, 600 nM) had a low Cq with a high ΔRn (green arrow); red arrow denotes primer concentrations that only had lowest Cq.

Figure S2.(B). The *R. amblyommatis* primer and probe optimization matrices (top) illustrate the optimal and lowest Cq (dark green) and highest Cq (dark red). In the amplification curves, the optimal Ramb primer concentration (150 nM, 300 nM) had a low Cq with a high ΔRn (green arrow); red arrow denotes primer concentrations that only had lowest Cq.

Figure S2.(C). The lone star tick primer and probe optimization matrices (top) illustrate the optimal and lowest Cq (dark green) and highest Cq (dark red). In the amplification curves, the optimal LST primer concentration (75 nM, 300 nM) had a low Cq with a high ΔRn (green arrow); red arrow denotes primer concentrations that only had lowest Cq.

Figure S2.(D). Average Cq value for each target from concentrations of 10^7 to 10^1 of plasmids from HEX-labeled *R.parkeri* (green), FAM-labeled *R. amblyommatis* (blue) and CY5-labeled LST (purple). Three plates tested in triplicates, shown here with efficiencies for each respective run next to image of Cq values.

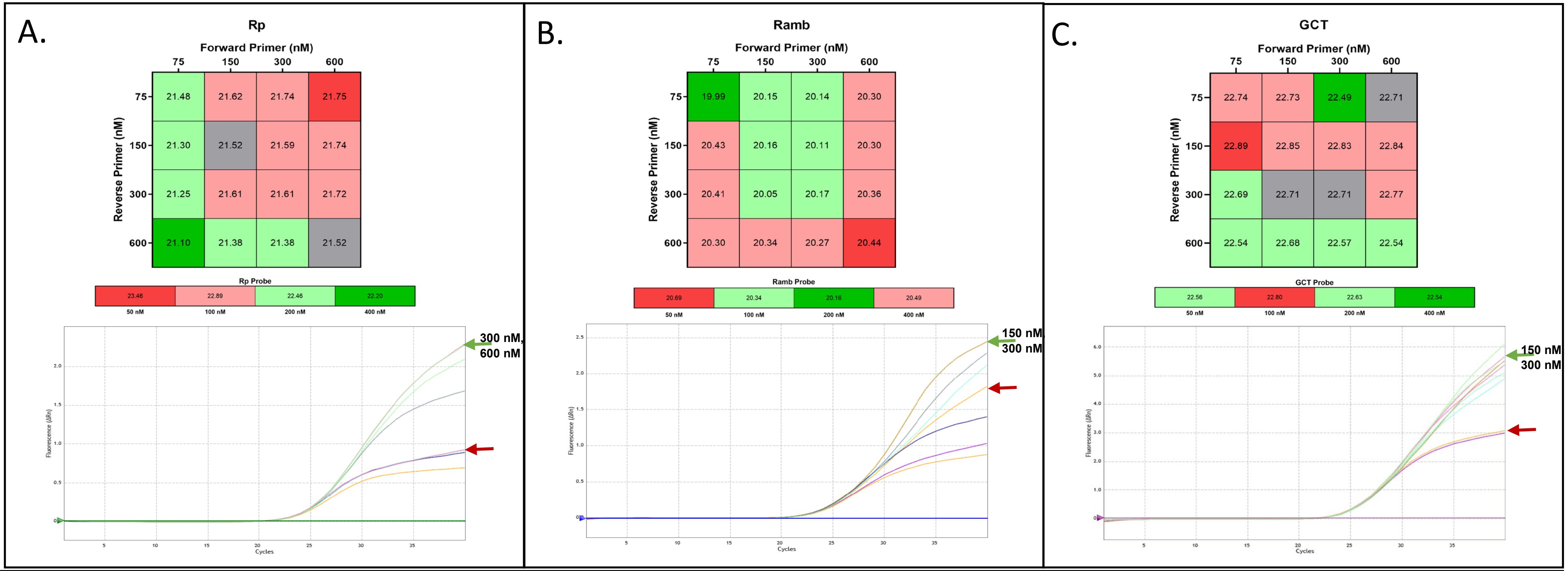

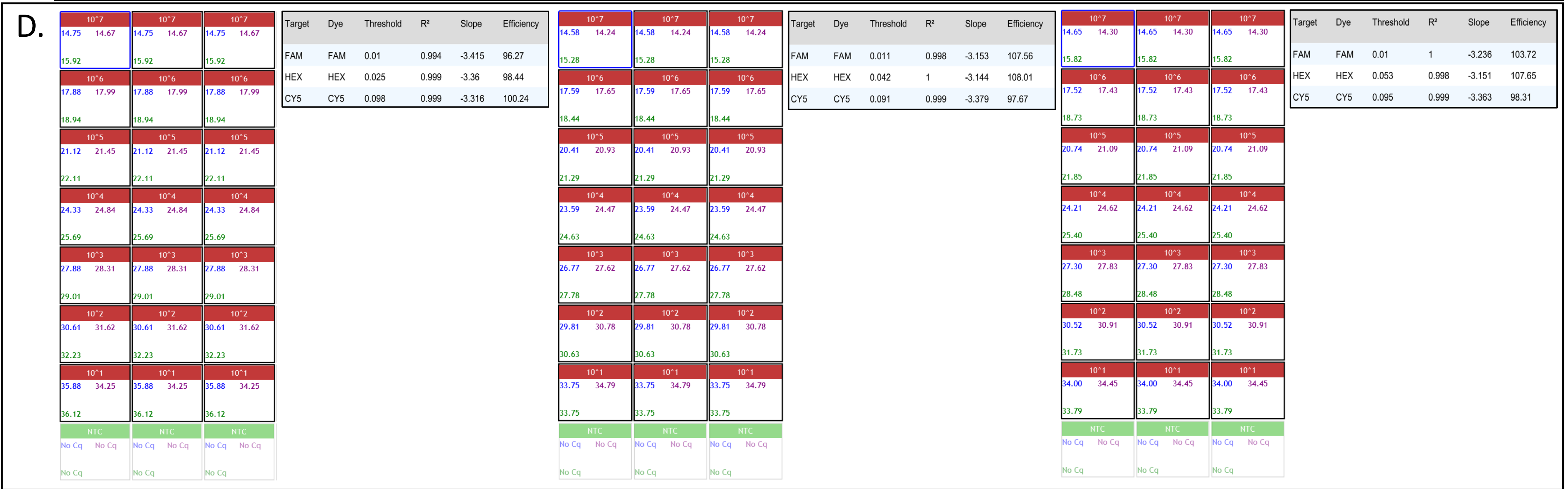

Target	Dye	Threshold	R²	Slope	Efficiency
FAM	FAM	0.01	0.994	-3.415	96.27
HEX	HEX	0.025	0.999	-3.36	98.44
CY5	CY5	0.098	0.999	-3.316	100.24

Target	Dye	Threshold	R²	Slope	Efficiency
FAM	FAM	0.011	0.998	-3.153	107.56
HEX	HEX	0.042	1	-3.144	108.01
CY5	CY5	0.091	0.999	-3.379	97.67

Target	Dye	Threshold	R²	Slope	Efficiency
FAM	FAM	0.01	1	-3.236	103.72
HEX	HEX	0.053	0.998	-3.151	107.65
CY5	CY5	0.095	0.999	-3.363	98.31

Figure S3.(A). The *R. parkeri* primer and probe optimization matrices (top) illustrate the optimal and lowest Cq (dark green) and highest Cq (dark red). Mean Cq values are gray. In the amplification curves, the optimal Rp primer concentration (300 nM, 600 nM) had a low Cq with a high ΔRn (green arrow); red arrow denotes primer concentrations that only had lowest Cq.

Figure S3.(B). The *R. amblyommatis* primer and probe optimization matrices (top) illustrate the optimal and lowest Cq (dark green) and highest Cq (dark red). . In the amplification curves, the optimal Ramb primer concentration (150 nM, 300 nM) had a low Cq with a high ΔRn (green arrow); red arrow denotes primer concentrations that only had lowest Cq.

Figure S3.(C). The Gulf Coast tick primer and probe optimization matrices (top) illustrate the optimal and lowest Cq (dark green) and highest Cq (dark red). Mean Cq values are gray. In the amplification curves, the optimal GCT primer concentration (150 nM, 300 nM) had a low Cq with a high ΔRn (green arrow); red arrow denotes primer concentrations that only had lowest Cq.

Figure S3.(D). Average Cq value for each target from concentrations of 10^7 to 10^1 of plasmids from HEX-labeled *R.parkeri* (green), FAM-labeled *R. amblyommatis* (blue), and CY5-labeled GCT (purple). Three plates tested in triplicates, shown here with efficiencies for each respective run next to image of Cq values.

Table S1. Complete list of reagents and resources.

REAGENT *or* RESOURCE	VENDOR	CATALOG NUMBER
Reagents		
Ampicillin	Sigma-Aldrich	A5354
BD Difco™ Dehydrated Culture Media: LB Broth, Miller	Fisher Scientific	DF0446-17-3
Blue/Orange Loading Dye, 6X	Promega	G1881
Brilliant Multiplex QPCR Master Mix	Agilent Technologies, Inc.	600553
DNeasy® Blood & Tissue Kit (250)	Qiagen	69506
Oxoid™ Agar Bacteriological	ThermoFisher Scientific	LP0011T
TOPO™ TA Cloning™ Kit for Sequencing, with pCR™4-	Invitrogen	K457502
TOPO™ Vector, One Shot™ TOP10 Chemically Competent	Invitrogen	K457502
E. coli, and PureLink™ Quick Plasmid Miniprep Kit	Invitrogen	K457502
Water, Molecular Biology Grade	Fisher Scientific	BP2819-1
100 bp DNA Ladder	Invitrogen	15628050
Materials		
AriaMx 96 Well Optical Plates	Agilent Technologies, Inc.	401494
Falcon® 100 mm x 15 mm Petri Dish	Corning	351029
Optical Cap, 8x Strip	Agilent Technologies, Inc.	401425
TempAssure 0.2 mL PCR 8-Tube Strips, Att. Optical Caps	USA Scientific	1402-3900
Instruments and Software		
Agilent AriaMx Real-Time PCR	Agilent Technologies, Inc.	
Agilent AriaMx Software (version 1.71)	Agilent Technologies, Inc.	https://www.agilent.com/
C1000 Touch Thermal Cycler	Bio-Rad	https://www.bio-rad.com/
GraphPad Prism (version 9.1.2)	GraphPad Software	https://www.graphpad.com/
NanoDrop™ One	ThermoFisher Scientific	701-058111
Powerpac 200 - For Electrophoresis & Blotting	Bio-Rad	https://www.bio-rad.com/
Qubit® 3.0 Fluorometer	ThermoFisher Scientific	Q33216
SnapGene®	GSL Biotech LLC	https://www.snapgene.com/
